# Elucidating the Immune-Related Mechanisms by Which Probiotic Strain *Lactobacillus casei* BL23 Displays Anti-tumoral Properties

**DOI:** 10.3389/fmicb.2018.03281

**Published:** 2019-01-11

**Authors:** Elsa Jacouton, Marie-Laure Michel, Edgar Torres-Maravilla, Florian Chain, Philippe Langella, Luis G. Bermúdez-Humarán

**Affiliations:** Micalis Institute, INRA, AgroParisTech, Université Paris-Saclay, Jouy-en-Josas, France

**Keywords:** probiotic, *Lactobacillus casei* BL23, anti-tumor, cancer, IL-2

## Abstract

We have recently described antitumor properties of *Lactobacillus casei* BL23 strain in both a mouse allograft model of human papilloma virus (HPV)-induced cancer and dimethylhydrazine-associated colorectal cancer. However, the mechanisms underlying these beneficial effects are still unknown. Interestingly, *in vitro* cellular models show that this bacterium is able to stimulate the production of high levels of IL-2. Because this cytokine has well-known antitumor properties, we decided to explore its role in the anti-cancer effects of BL23 using the HPV-induced cancer model. We found a negative correlation between IL-2 and tumor size confirming the necessity of IL-2 to protect from tumor development. Then, we blocked IL-2 synthesis using neutralizing monoclonal antibodies in mice that were challenged with lethal levels of tumor cells; this led to a significant reduction in the protective abilities of BL23. Next, we used a genetically modified strain of *Lactococcus lactis* to deliver exogenous IL-2 to the system, and in doing so, we were able to partially mimic the antitumor properties of BL23. Additionally, we showed the systemic role of T-cells in tumor protection through a negative correlation between tumor size and T-cells subpopulations and an increasement of BL23-specific local Foxp3 levels in tumor-bearing mice. Finally, we observed a negative correlation between tumor size and NK+ cells, but local recruitment of NK cells and cytotoxic activity appeared specific to BL23 treatment. Taken together, our data suggest that IL-2 signaling pathway plays an important role in the anti-tumoral effects of probiotic strain *L. casei* BL23. These results encourage further investigation in the use of probiotic strains for potential therapeutic applications to clinical practice, in particular for the treatment of colorectal cancer. Furthermore, our approach could be extended and applied to other potential beneficial microorganisms, such as gut microbiota, in order to better understand the crosstalk between microbes and the host.

## Introduction

The Food and Agriculture Organization of the United Nations World Health Organization has defined probiotics as “live microorganisms which, when administered in adequate amounts, confer health benefits on the host” (FAO/WHO, 2002). A growing body of evidence suggests that probiotics can reinforce natural defenses, protect against gastrointestinal disorders, and enhance either innate or adaptive immunity. Indeed, it has been shown that probiotics can induce dendritic cell (DC) maturation (Delcenserie et al., 2008), enhance natural killer (NK) cell cytotoxicity (Takagi et al., 2001), and upregulate cytokine secretion (Delcenserie et al., 2008; Azcarate-Peril et al., 2011).

Lactobacilli, which belong to the group of lactic acid bacteria (LAB), feature prominently among putative probiotics. In addition, some strains are naturally present in human mucosal surfaces (e.g., vagina and gastrointestinal tract), where they make up a small portion of the microbiota and are thus considered commensals (Martin et al., 2014). Some strains of *Lactobacillus* have been used for centuries in the preservation and production of fermented foods (e.g., yogurt and cheese; (Bernardeau et al., 2008)) and are thus “Generally Recognized As Safe” by the U.S. Food and Drug Administration. These microorganisms exert beneficial health effects on the host, mainly by modulating immune response (Martin et al., 2013). In addition, a number of studies have demonstrated that consumption of probiotics, including lactobacilli, may play a preventive role in the onset of different types of cancer (Kato et al., 1994; Aragón et al., 2015; Hu et al., 2015). It has also been reported that some strains of *Lactobacillus* can induce DC maturation and differentiation of the Th1 immune response, which is important for tumor inhibition (Cai et al., 2016). However, despite the number of studies that have shown anti-cancer effects of different strains of *Lactobacillus* (Khazaie et al., 2012; Konishi et al., 2016; Lenoir et al., 2016), the precise host molecular mechanisms of these antitumor properties remain unclear.

Many studies have proposed that the effects of probiotics might involve: (i) modification of gut pH, (ii) an increase in the production of short-chain fatty acids (SCFAs; e.g., acetate, propionate, and butyrate), (iii) antagonistic activity against pathogens through the production of antimicrobial compounds (such as bacteriocins), or (iv) competition with pathogens for available nutrients, receptors, and growth factors (for review see refs. in (Kahouli et al., 2013)). Also, oral administration of LAB has been shown to effectively reduce DNA damage induced by chemical carcinogens, in particular in different animal models of colorectal cancer (CRC; Uccello et al., 2012; Kahouli et al., 2013). However, in all of these studies the proposed antitumor effects are associated with oral intake of the probiotic. Despite this, we recently demonstrated anticancer effects of the dairy strain BL23 of *L. casei* in a mouse allograft model of human papilloma virus (HPV)-induced cancer when the probiotic was administered intranasally (Lenoir et al., 2016). In addition, we also showed that oral administration of this probiotic strain was able to reduce the severity of 1,2-dimethylhydrazine (DMH)-associated CRC (Lenoir et al., 2016). Although the precise mechanisms of this antitumor effect were not clearly established, we hypothesized that *L. casei* BL23 can activate antitumor immunity and retard tumor growth. Indeed, in the model of DMH-associated CRC we showed that the defense against cancer demonstrated by *L. casei* BL23 was associated with the modulation of T-cells toward a Th17-biased immune response, accompanied by the expression of regulatory cytokines (e.g., IL-6, IL-17, IL-10, and TGF-β). In this study, we decided to further examine the mechanisms of this defense by investigating the host cell-signaling pathway associated with the antitumor properties of *L. casei* BL23, using the HPV-induced cancer model. In particular, we sought to evaluate the potential involvement of the IL-2-driven immune response in the antitumor effects of *L. casei* BL23. This cytokine and its associated pathway have been linked with positive effects in cancer therapy (Rosenberg et al., 1985), in particular in HPV-associated tumor cells (Casana et al., 2002). In addition, *L. casei* BL23 has been reported to induce high levels of this cytokine in both *in vivo* (Dorostkar et al., 2016) and *in vitro* models (including DCs and monocytes; personal observations).

## Materials and Methods

### Bacterial Strains and Growth Conditions

*Lactobacillus casei* BL23 (Acedo-Felix and Perez-Martinez, 2003; Maze et al., 2010) was grown in MRS medium (Difco, KS, Unites States) at 37°C under static conditions. *L. lactis* MG1363 (Gasson, 1983) and recombinant *L. lactis* NZ9000, which secretes murine IL-2 (strain LL-IL2, Supplementary Data and Supplementary Figure S1), were grown in M17 medium (Difco, KS, United States) supplemented with 0.5% glucose (GM17) at 30°C under static conditions. Strain LL-IL2 was maintained with chloramphenicol at a concentration of 10 μg/ml. For IL-2 production, the strain was grown to an optical density at 600 nm (OD_600_) of 0.6, then induced with 10 ng/ml of nisin (Sigma) for 1 h (as previously described in (Bermudez-Humaran et al., 2003)). LL-IL2 culture extraction and immunoblotting assays were performed as described in (Bermudez-Humaran et al., 2015) using murine IL-2 antibodies (Peprotech, see Supplementary Data).

### TC-1 Cell Line

The mouse (C57BL/6) lung tumor line TC-1 (kindly provided by Dr. T.C. Wu, Johns Hopkins University, Baltimore, MD, United States in 2000) was grown in RPMI (Roswell Park Memorial Institute) medium 1640 (Lonza, Switzerland) supplemented with 10% heat-inactivated fetal calf serum (FCS), 50 U/ml penicillin, and 50 U/ml streptomycin (Lonza, Levallois-Perret, France) in a 5% CO_2_ atmosphere. The TC-1 cell line was generated by transduction with a retroviral vector that express HPV-16 E6/E7 plus a retrovirus expressing activated c-Ha-*ras* (Lin et al., 1996). The authenticity of TC-1 cells was regularly controlled in our laboratory, first by the addition in each experiment of both 0.4 mg/ml G418 (Geneticin) (Sigma-Aldrich, St. Louis, MO, Unites States) and 0.2 mg/ml hygromycin (Sigma-Aldrich, St. Louis, MO, United States) to maintain the selective pressure for HPV-16 E6/E7 and c-Ha-*ras*, and second, by confirming the presence of the HPV-I6 E7 gene by PCR (once every 6 months). Once a month, TC-1 cells were confirmed to be mycoplasma-free with a PCR-based detection test.

### Mice

Specific pathogen-free C57BL/6 mice (females, 6–8 weeks old; Janvier SAS, St. Berthevin, France) were housed in a pathogen-free isolator (four mice per cage) under sterile conditions in 12-h light cycles in the animal facilities of the National Institute of Agricultural Research (IERP, INRA, Jouy-en-Josas, France). Animals were supplied with water and fed *ad libitum* (normal chow: R 03–40, SAFE). Temperature and moisture were carefully controlled. Mice were observed once a day to ensure their welfare. All protocols were carried out in accordance with the institutional ethical guidelines of the ethics committee COMETHEA (Comité d’Ethique en Expérimentation Animale of the Centre INRA of Jouy-en-Josas and AgroParisTech), which approved this study.

### Production of IL-2 by Bone Marrow Dendritic Cells (BMDCs)

Bone Marrow Dendritic Cells (BMDCs) were generated from bone marrow cells isolated from femurs and tibias of specific pathogen-free C57BL/6 mice (males, 4–6 weeks old; Janvier SAS, St. Berthevin, France). BM cells were separated via either Ficoll-Paque density separation (Lympholyte-Mammal, Cedarlane) or 75 μm strainer and cultured at ∼1 × 10^6^ cells/well using 24-well plates and DMEM medium (Sigma-Aldrich, St. Louis, MO, United States) at 37°C with 10% CO_2_. The medium was supplemented with 10% heat-inactivated FCS (Lonza, Switzerland), 50 mM 2-mercaptoethanol (Sigma-Aldrich, St. Louis, MO, United States), 1 mM glutamine (Lonza, Switzerland), 50 U/ml penicillin (Lonza, Switzerland), 50 g/ml streptomycin (Lonza, Switzerland), and 20 ng/ml of recombinant murine granulocyte-macrophage colony-stimulating factor (GM-CSF; Peprotech, France). Freshly prepared medium was added every 3 days. After 10 days, BMDC cultures (higher CD11c expression confirmed by FACS analysis) were treated with *L. casei* BL23 or *L. lactis* MG1363 at a dose of 1:1, 1:40 and 1:100 CFUs per BMDC. After 24 h of incubation, supernatant samples were recovered and examined with ELISA (Mabtech) for the presence of IL-2.

### Bacterial Administration in TC-1-Challenged Mice

Groups of mice (*n* = 8) were intranasally (*i.n.*) administered 1 × 10^9^ colony-forming units (CFU) of either *L. casei* BL23, *L. lactis* MG1363, or recombinant LL-IL2, which were suspended in 10 μl of PBS and administered using a micropipette after intraperitoneal anesthetization. All cultures were washed three times in PBS before being suspended for administration. Each mouse received 5 μl of the solution in each nostril on days –35, –21, and –7 (Supplementary Figure S2A). Control mice received identical quantities of PBS (i.e., 10 μl). Mice were challenged 7 days after the final bacteria administration (D0) by subcutaneous (*s.c.*) injection in the right rear flank with 5 × 10^4^ TC-1 cells in 100 μl of sterile PBS. The dimensions of the tumor at the site of injection were measured every week in two perpendicular directions with a caliper, and tumor volume was estimated as (length × width^2^)/2 (Bermudez-Humaran et al., 2005). Mice were sacrificed by vertebral dislocation at D28 (Supplementary Figures S2A, S2B).

For IL-2 blocking experiments, mice received (once every 2 days) intraperitoneal (*i.p.*) injections of 100 μl of IL-2-neutralizing monoclonal antibodies (mAb 500-M127; Peprotech, France) prepared in PBS at 50 μg/ml (Supplementary Figure S2A).

In a final experiment, mice were administered *s.c.* 1 × 10^7^ CFU of *L. casei* BL23 (*n* = 8) or PBS (*n =* 4) once every 2 days after challenge with a tumor (Supplementary Figure S2B).

### *Lactobacillus casei* BL23 Localization After *i.n.* Administration

*Lactobacillus* *casei* BL23 was stained with syto 9 (Live/Dead Baclight Bacterial Viability Kit, ThermoFisher Scientific, France) during 15 min at room temperature in the dark according to the manufacturer’s instructions. Then, four mice were administered either with 1 × 10^9^ CFU of stained *L. casei* BL23, unstained *L. lactis* MG1363 or equivalent volume of PBS. Broncho-Alveolar Liquid (BAL), Nasal-Associated Lymphoïd Tissue (NALT) and stomach content were collected 30 min, 24 h and 48 h after *i.n.* administration as previously described in Medaglini et al. (2006). NALT and stomach were mechanically crushed in 300 μl or 1 ml of PBS, respectively, prior to be filtered through 40 μm cell stainer. Stained *L. casei* BL23 was detected using a flow cytometer “(Accuri C6)”.

### Immune Cells Analysis Using Flow Cytometry

Spleens were collected and mononuclear cells isolated via gentle extrusion of the tissue through a 50-μm-mesh nylon cell strainer (BD). Cells were resuspended in DMEM medium supplemented with 10% FCS, 2 mM L-glutamine, 50 U/mg penicillin, and 50 U/mg streptomycin. Erythrocytes were lysed with red-blood-cell lysing buffer (Sigma-Aldrich, St. Louis, MO, United States). Cells collected at D28 were first stained with eFluor 506-labeled fixable viability dye (eBioscience), and a surface staining was then performed with APC-eFluor780-labeled anti-CD3 (145-2C11), PECy7-labeled anti-NKp46 (29A1.4), PE-labeled anti-CD4 (RM4–5), FITC-labeled anti-Foxp3 (FJK-16s) and Brillant Violet 605-labeled anti-CD8α (53–6.7) antibodies. Flow cytometry analyses were carried out using a BD-Fortessa machine. Data were analyzed using FlowJo software (version X10.0.7).

### Analysis of Gene Expression Profiles in Tumor Sections

Tumor samples were stored in RNAlater (Sigma Aldrich, St. Louis, MO, United States) at –80°C following animal sacrifice. Total RNA was extracted using the RNeasy mini-kit (Qiagen, Courtaboeuf, France) according to the manufacturer’s recommendations. RNA concentration was measured using a NanoDrop spectrophotometer (NanoDrop Technologies, Wilmington, DE,United States) and 1 μg was used to synthesize cDNA using the High Capacity cDNA Reverse Transcription kit (Applied Biosystems, Foster, CA, United States), following the manufacturer’s instructions. The qPCR mixture contained Taqman probes (Life Technologies, France; primers are described in Table 1) and reactions were carried out according to the manufacturer’s instructions using an ABI Prism 7700 thermal cycler (Applied Biosystems, Foster, CA, United States) in a reaction volume of 20 μl. To quantify and normalize the expression data, we used the ΔCt method with β-actin as a reference gene.

**Table 1:** Sequence of Taqman primers used in this study.

Probes	Targets
Mm00446171_m1	mCD3/CD247
Mm00439860_m1	mIL-2/Itk
Mm00607939_s1	mβ-Actin
Mm01337324_g1	mNKp46/Ncr1
Mm00475162_m1	mFoxp3
Mm01182107_g1	mCD8α


### Cytotoxic Activity of NK Cells

Natural killer (NK) cells were isolated using the NK cell isolation kit II, mouse (Miltenyl Biotec, France), following the manufacturer’s instructions, from mice that had been treated with either *L. casei* BL23 or *L. lactis* MG1363. TC-1 cells were grown as described above and cultivated in petri dishes at 5 × 10^5^ cells per dish for 2 days. NK cells were then added at a 1:20 ratio and cultivated for 5 days before microscopic observations (Zeiss microscope Axio observer Z1) as described in Ribelles et al. (2013).

### Statistical Analysis

#### Data Were Analyzed With Prism Software (Version 5)

All data were displayed as mean ± s.e.m. Comparisons between two groups were performed with *T*-test followed by Mann–Whitney post-test. Groups of three or more animals were analyzed by one-way analysis of variance (ANOVA). Mice treated with *L. casei* BL23 and displaying tumors were excluded from the analysis.

## Results

### *Lactobacillus casei* BL23 Has a Protective Effect Against Tumors in the TC-1 Allograft Model of HPV-Induced Cancer

**FIGURE 1:**
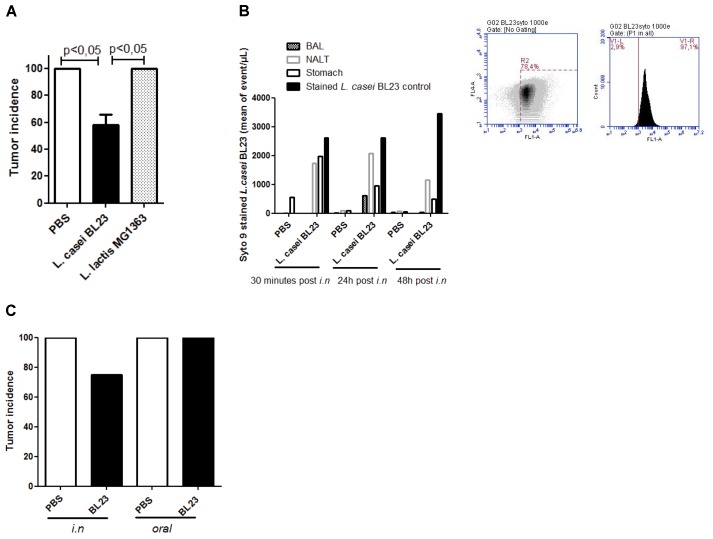
*Lactobacillus casei* BL23 protection against TC1-induced tumors: influence of the route of administration **(A)** Tumor incidence in the TC-1 mouse allograft model of HPV-induced cancer. Mice were intranasally administered either *L. casei* BL23, *L. lactis* MG1363, or sterile PBS three times (bacteria at a concentration of 1 × 10^9^ CFU). A week following the final administration, mice were challenged with 5 × 10^4^ TC-1 tumor cells (*n =* 3 independent *in vivo* experiments at *n =* 8 mice, *p* < 0.05, ANOVA). **(B)** *L. casei* BL23 detection after *i.n.* administration. Mice were *i.n.* administered with stained *L. casei* BL23 (*n =* 4 mice per group), stained *L. casei* BL23 (dilution by 10 after 24 and 48 h of storage at 4°C in the dark) was used as positive control. **(C)** Comparison of tumor incidence between oral and *i.n.* administration protocol (*n =* 8 mice). Data are represented as the mean of each group ± SEM.

We recently reported that *L. casei* BL23 displayed a protective effect in a mouse allograft model of HPV-induced cancer (Lenoir et al., 2016). Here we first validated these prior observations using the same conditions (e.g., bacteria dosage, *i.n.* administration, schema of administration; Supplementary Figure S2A) and we compared these results to those obtained with *L. lactis* MG1363, a LAB strain for which no positive effect has been reported in the HPV-induced cancer model (Bermudez-Humaran et al., 2005). Our results showed that *L. casei* BL23 had a significant protective effect against tumor onset at day 28 (the day on which animals were sacrificed): only 58% (14/24) of mice that had been administered *L. casei* BL23 developed tumors, compared to 100% of mice receiving either PBS or *L. lactis* MG1363 (Figure 1A). These results confirm the absence of any positive effect of *L. lactis* MG1363, which is consistent with previous reports (Bermudez-Humaran et al., 2005; Cortes-Perez et al., 2007). We then determined bacterial location after *i.n.* administration using Syto 9 bacterial staining. We mainly found *L. casei* BL23 in NALT and stomach (Figure 1B) 24 h and 48 h after administration. Despite a higher *L. casei* BL23 concentration was observed (2069 events/μl as mean in NALT vs. 950 events/μl as mean in stomach) in NALT than in stomach 24h post inoculation; we compared the effect on tumor onset in function of the route of bacterial administration (oral vs. *i.n.*). We confirmed previous data obtained with recombinant bacteria showing no protective effect following oral bacterial administration (Figure 1C).

### Tumor Size Was Negatively Correlated With Levels of IL-2

Several studies have investigated IL-2 for potential use in tumor growth inhibition. So we determined the local IL-2 expression (in tumor sections) of mice bearing tumors by qPCR. Interestingly, we observed a significant negative correlation between tumor size and local IL-2 levels in tumor samples (*r*^2^ = –0.75, *p* = 0.0008, Supplementary Figure S3A), suggesting a positive role for this cytokine to treat tumors.

**FIGURE 2:**
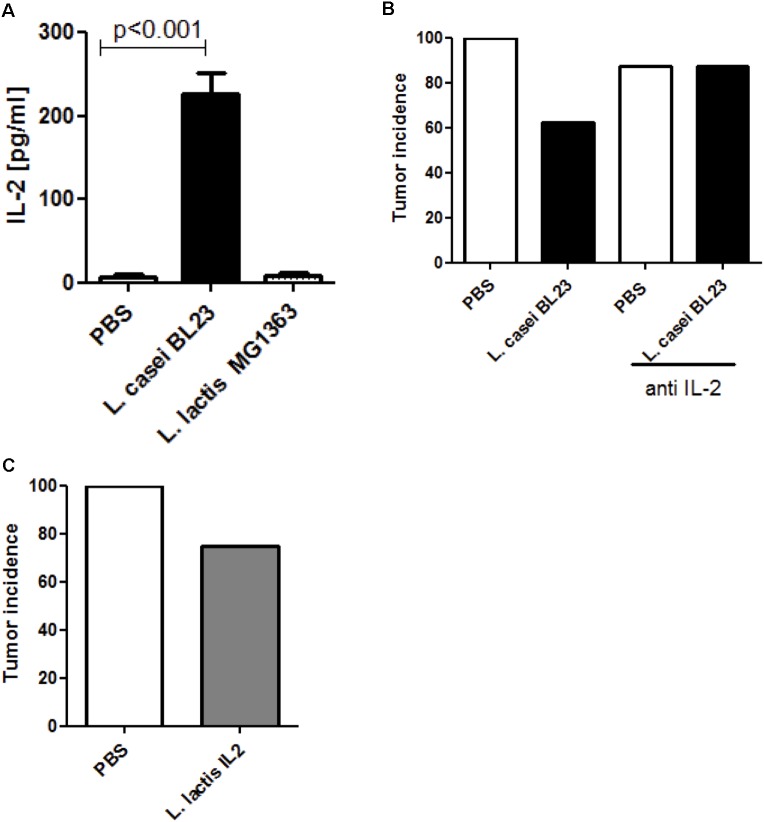
IL-2 involvement in tumor protection. **(A)** IL-2 production by murine DCs (*n* = 3 independent experiments, *p* < 0.001, ANOVA). BMDCs were exposed to bacteria at MOI = 100 for 24 h. **(B)** Tumor incidence. Mice were administered PBS or *L. casei* BL23 in the presence or absence of anti-IL-2 mAb. **(C)** Effect of *L. lactis*-IL2 treatment on protection against tumors. Data are represented as the mean of each group ± SEM (*n* = 8 mice per group).

To assess if *L. casei* strain BL23 could exerted its anti-tumor effects *via* IL-2, we first investigated its abilities to induce IL-2 expression in an *in vitro* model of BMDCs. Specifically, BMDCs were treated with either *L. casei* BL23 or *L. lactis* MG1363 for 24 h at MOI 100. As shown in Figure 2A, the presence of *L. casei* BL23 specifically induced IL-2 expression while *L. lactis* MG1363 did not. Additionally, we determined an IL-2 dose response in presence of *L. casei* BL23. Our results showed that *L. casei* BL23 significantly increased IL-2 production at MOI 1:40 and 1:100 (Supplementary Figure S1B).

We next determined the role of IL-2 in the antitumor effects of *L. casei* BL23 *in vivo*. For this, we blocked IL-2 production in mice via *i.p.* injections of IL-2-neutralizing mAb 2 days before and 2 days after *i.n.* bacterial treatments and then once every 2 days following *s.c.* TC-1 challenge (Supplementary Figure S2A). As shown in Figure 2B, 62.5% of mice (5/8) treated with *L. casei* BL23 developed tumors over the 28-day test period compared to 100% of control mice (8/8) treated with PBS. Conversely, mice treated with anti-IL-2 mAb lost the protective effect of *L. casei* BL23 (Figure 2B): 87.5% of mice (7/8) that were treated with both *L. casei* BL23 and anti-IL-2 mAb developed tumors, and the same result was found with control mice receiving PBS.

In order to confirm that IL-2 had a protective effect against tumor development in our model, mice were *i.n.* administered with a recombinant strain of *L. lactis* which secretes murine IL-2 (LL-IL2, Supplementary Figure S1A, and Supplementary Data). We confirmed IL-2 production via ELISA prior to administration of the recombinant strain to mice (Supplementary Figure S1C). As shown in Figure 2C, the administration of LL-IL2 prevented tumor development in 25% of mice (2/8), while tumors were observed in 100% of PBS-treated mice.

Altogether, these data suggest that the IL-2 signaling pathway plays an important role in the ability of BL23 to control tumor development in the HPV-induced model of cancer.

### Both Systemic and Local T Immune Cells Are Crucial to Protect Against Tumor Growth

**FIGURE 3:**
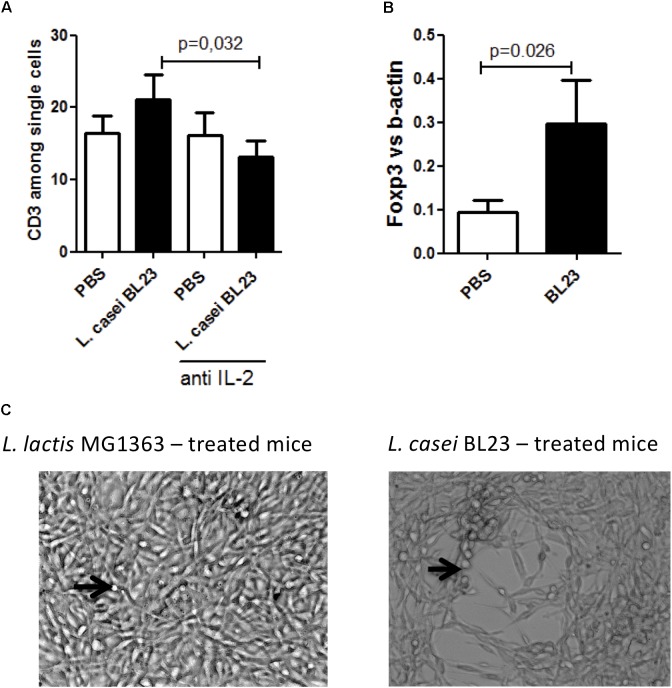
A potent role of immune cells in *L. casei* BL23 tumor protection after i.n adminsitration. **(A)** CD3+ cells level in spleen (*n* = 8 mice per group; *p* = 0.0032, *T*-test followed by Mann–Whitney post-test)). **(B)** Foxp3 level in tumor section (*n* = 8 mice per group; *p* = 0.026, *T*-test followed by Mann–Whitney post-test). **(C)** *In vitro* cytotoxic activity of NK cells isolated from mice treated with (right panel) *L. casei* BL23 or (left panel) *L. lactis* MG1363. TC-1 cells, which formed a dense and well-adhering layer in the presence of NK from *L. lactis* MG1363-administered mice, almost completely disappeared in the presence of NK from mice treated with *L. casei* BL23. TC-1 cells were incubated with NK cells at a ratio of 1:20 for 5 days. Black arrows indicate NK cells.

To go deeper in the analysis of the immune response related to tumor inhibition observed with BL23, splenocytes and tumor sections were harvested and immune cell populations were assessed using flow cytometry and qPCR, respectively. We first analyzed the systemic expression of immune T cells subpopulation. A negative correlation between tumor size and the abundance of both CD3+ (*r*^2^ = –0.87, *p* < 0.0001, Supplementary Figure S3B), CD8+ (*r*^2^ = –0.84, *p* < 0.0001, Supplementary Figure S3C), CD4+ (*r*^2^ = –0.85, *p* < 0.0001, Supplementary Figure S3D) and FOXP3+ (*r*^2^ = –0.88, *p* < 0.0001, Supplementary Figure S3E) cells was observed. Furthermore, anti-IL-2 treatment had no effect on the proportion of systemic CD3+ cells compared to PBS treatment. However, mice treated with *L. casei* BL23 exhibited significantly reduced levels of CD3+ cells when anti-IL-2 antibodies were also administered (Figure 3A).

In addition, we only observed a negative correlation between tumor size and local CD3+ cells (*r*^2^ = –0.84, *p* = 0.002, Supplementary Figure S3F). However, mice bearing tumor and treated with *L. casei* BL23 exhibited significantly higher local Foxp3 level (Figure 3B).

Interestingly, we also detected a positive correlation between local levels of CD3+ cells and IL-2 in tumor-bearing mice (*r*^2^ = –0.83, *p* < 0.0001, Supplementary Figure S3G).

These data highlight the crucial role of both systemic and local T-immune response after i.n. administration of BL23 to combat tumor growth, and point out the important role of IL-2 pathway in these anti-cancer effects.

### NK Cells Mediated a Cytotoxic Effect of *L. casei* BL23 Toward Cancer Cells

Because of the well-known role of IL-2 in NK cell proliferation and the cytotoxic activities of these cells on tumor development (Takagi et al., 2001), we assessed the systemic expression of NK cells. A negative correlation between tumor size and NKP46+ cells was found confirming the necessity of NK cells in tumor protection (*r*^2^ = –0.86, *p* < 0.0001, Supplementary Figure S3H).

More interestingly, there was a significant negative correlation between local NKp46 expression and tumor size (*r*^2^ = –0.94, *p* = 0.016, Supplementary Figure S3I). This correlation was specific to *L. casei* BL23 treatment since no correlation was observed between NK cells and tumor size in control PBS-treated mice (data not shown).

We further analyzed cytotoxicity activity of NK on TC-1 cells *in vitro*. For this, NK cells were isolated from mice that had been treated *i.n.* with either *L. lactis* MG1363 (negative control) or *L. casei* BL23, and cultivated in the presence of TC-1 cells for 5 days. As shown in Figure 3C, TC-1 cells that were exposed to NK cells from *L. casei* BL23-treated mice exhibited a larger lysis area than those exposed to NK cells isolated from *L. lactis* MG1363-treated mice. These data suggest that, unlike *L. lactis* MG1363, *L. casei* BL23 induced the recruitment of NK cells with high potent cytotoxicity toward tumor cells.

### Therapeutic *s.c.* *L. casei* BL23 Administration Resulted in Reduced Tumor Volume

**FIGURE 4:**
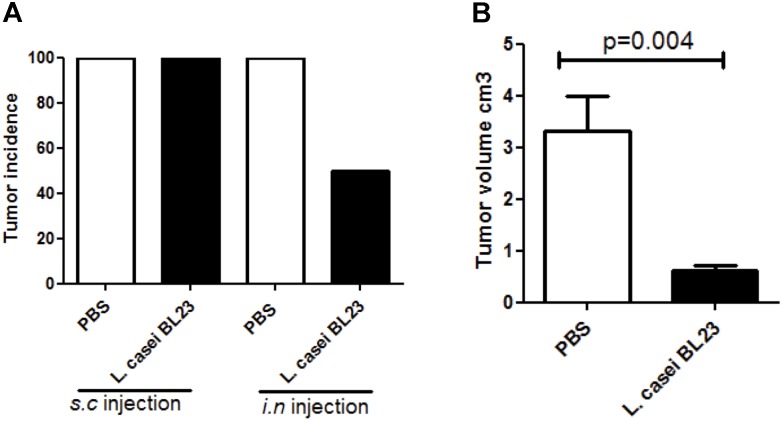
Assessment of therapeutic *s.c.* introduction of *L. casei* BL23 at the site of TC-1 injection. **(A)** Tumor incidence, **(B)** tumor volume. Mice were challenged with TC-1 tumors and, beginning 24h later, received *s.c*. injections of bacteria every 2 days. Data are represented as the mean of each group ± SEM (*n =* 4 to 8 mice, respectively, for PBS or BL23 treated group; *p =* 0.004, *T*-test followed by Mann–Whitney post-test).

It is well known that some species of anaerobic bacteria can selectively migrate after intravenous injection and grow in the hypoxic regions of solid tumors (Kimura et al., 1980). As *L. casei* BL23 is a facultative anaerobic Gram-positive bacterium, we cannot discard the possibility that this strain has a local effect in the tumor environment, especially in regards of the observed local immune response. To investigate this, we performed local *s.c.* administration of *L. casei* BL23 post-TC-1 injection (Supplementary Figure S2B). For this, mice were first challenged with tumor cells and, 1 day later, we administered *s.c.* bacteria at the same injection site. Our results revealed that, in contrast to the prophylactic effect of *i.n.* treatment with *L. casei* BL23 prior to tumor challenge, *s.c.* therapeutic administration had no effect on tumor incidence (Figure 4A), however, a significant reduction in tumor size was observed (Figure 4B).

**FIGURE 5:**
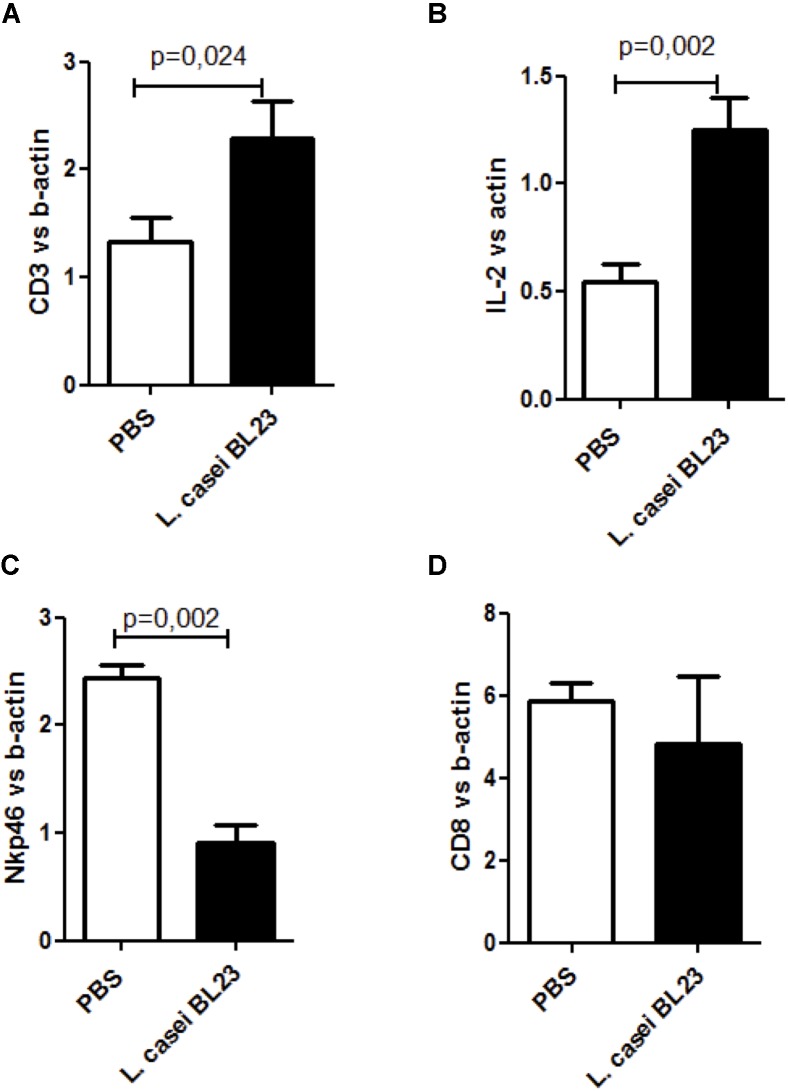
Local immune response induced by *L. casei* BL23 following *s.c.* administration. **(A)** CD3 level (*p =* 0.024, *T*-test followed by Mann–Whitney post-test), **(B)**, IL-2 level (*p =* 0.002, *T*-test followed by Mann Whitney post-test), **(C)** Nkp46 level (*p =* 0.002, *T*-test followed by Mann–Whitney post-test), and **(D)** CD8 level in tumor sections (ns, *T*-test followed by Mann–Whitney post-test). Data are represented as the mean of each group ± SEM (*n =* 4 to 8 mice, respectively, for PBS or BL23 treated group).

Furthermore, as shown in Figure 5, *s.c.* therapeutic administration of *L. casei* BL23 increased CD3 (Figure 5A) and IL-2 levels (Figure 5B) and reduced Nkp46 levels (Figure 5C) in tumor sections. No regulation was observed for CD8+ cells (Figure 5D).

These results suggest that *L. casei* BL23 can have a beneficial effect in the HPV-induced cancer model following both *i.n.* and local administration (*s.c.*). This highlights the possibility of using *L. casei* BL23 not only as a preventive agent but also as a form of therapy to decrease tumor size.

## Discussion

Lactic acid bacteria are widely used in the food industry; they are safe (having been widely consumed by humans for centuries in fermented foods) and can confer many beneficial effects on host health. Indeed, LAB have been linked with several probiotic activities, including the stimulation of the immune system (for a review see (Martin et al., 2013)). In addition, some LAB species are members of healthy human gut microbiota. Recently, several studies have reported the ability of certain LAB strains to inhibit tumor development (Khazaie et al., 2012; Konishi et al., 2016; Lenoir et al., 2016). Among the potential anti-tumoral mechanisms of LAB, two of the most promising are the modulation of the immune response and the induction of cellular apoptosis. For instance, two strains of *L. casei* are able to decrease tumor cell proliferation and enhance apoptosis in allograft models of colorectal cancer (Lee et al., 2004; Baldwin et al., 2010; Konishi et al., 2016). Similarly, oral administration of an *L. casei* strain reduces the onset of chemically induced tumors via the stimulation of IL-12 or NK-cell cytotoxicity mechanisms (Takagi et al., 2001, 2008). Furthermore, our team recently demonstrated the protective effects of the probiotic strain *L. casei* BL23 in different mouse models of cancer, including colorectal-associated cancer (CAC) and the TC-1 allograft model (Lenoir et al., 2016). In the CAC model, *L. casei* BL23 was linked with reduced expression of pro-inflammatory cytokines, but the molecular and cellular mechanisms involved in TC-1 cancer prevention were not elucidated.

First, we investigated the role of the bacterial route of administration: *i.n.* vs. oral. We effectively showed that stained *L. casei* BL23 was mainly founded in NALT after *i.n.* administration with a posterior transit to the stomach. Interestingly, oral administration of BL23 which resulted in a local localization of the bacterium (i.e., digestive tract, did not protect mice from tumor onset, confirming that *i.n.* administration of BL23 is crucial in the process of tumor protection in our animal model. Surprisingly, we failed to locate administered bacteria in BAL samples as previously described for other LAB (Grangette et al., 2001), however, in that study, their analyses were performed later (10 days after instillation) and we cannot exclude that we examined bacterial location too early for BAL detection. However, NALT has been described as an important tissue not only for mucosal but also for systemic immune response stimulation (Vintini and Medina, 2014) (Medaglini et al., 2006). Indeed, Vintini et al. reported a specific T-cell response after administration of another *L. casei* strain in NALT and serum samples. So, we cannot rule out a NALT-specific immune response induced by the presence of bacteria after instillation. To test this hypothesis, more investigations concerning NALT-related sequential immune response after BL23 inoculation are necessary.

IL-2 is a polyvalent cytokine with effects on the activation and regulation of immune cells, and is expressed by both lymphoid and non-lymphoid cells (Hoyer et al., 2008). For 15 years, several works have investigated IL-2 for potential use in tumor growth inhibition (Den Otter et al., 1995; Den Otter et al., 1998; Casana et al., 2002; Kusnierczyk et al., 2004). In this context, we hypothesized that the anti-tumoral activity associated with *L. casei* BL23 in our allograft model could be due to IL-2 regulation and the subsequent immune response. Our results showed that, *L. casei* BL23 is able to boost IL-2 secretion *in vitro*. Additionally, we observed that mice with low level of local IL-2 (in tumor sections) presented larger tumors. Strikingly, when mice were treated with IL-2-neutralizing mAb prior to both *i.n.* administration with *L. casei* BL23 and *s.c.* challenge with TC-1 cells, *L. casei* BL23 failed to protect against TC-1 tumor onset. Instead, administration of IL-2 via a recombinant strain of *L. lactis* (LL-IL2) partially mimicked the protective phenotype, although not up to the levels observed with *L. casei* BL23. These results highlight the key, but not exclusive, role of IL-2 signaling in *L. casei* BL23-associated protection against tumors in the mouse allograft model of HPV-induced cancer.

To further decipher the BL23 specific immune response, we first analyzed it at a systemic level. We observed that CD3+ (a marker of T cells), CD8+ (a marker of cytotoxic T-cells), CD4+ (a marker of TH cells) and Foxp3+ (a marker of regulatory T cells) were negatively correlated with tumor size suggesting that most important is the T-cell immune response, lower is tumor grow. We showed that in presence of anti-IL-2, mice which are not protected from tumors had also lower systemic CD3+ cells. In addition, when we specifically investigated the local immune response, we observed a specific up-regulation of Foxp3 in mice treated with BL23 and a negative correlation between tumor size and levels of CD3+ Moreover, in tumor-bearing mice, IL-2 and CD3+ levels were positively correlated with each other, suggesting that in mice with large tumors, CD3+ levels may decrease as a result of IL-2 dependence. These results highlight the efficacy of both T immune cells and IL-2 levels as good prognosis markers in tumor-bearing mice.

Furthermore, when analyzing tumor sections from mice treated with *L. casei* BL23, we detected a specific, negative correlation between NKp46 cell abundance and tumor size. Intrigued by the positive regulation of local and systemic NK cells in mice, we analyzed the effect of *L. casei* BL23 on the cytotoxic activity of NK cells and determined that NK cells from mice treated with *L. casei* BL23 displayed higher lysis activity against TC-1 cells than NK cells from control mice did.

These data are consistent with the results of multiple studies that highlight the role of T-cell subpopulations and NK cells in improved survival for patients suffering from cervical adenocarcinoma as well as in higher resistance in TC-1-challenged mice (Bermudez-Humaran et al., 2005; Brulet et al., 2007; Punt et al., 2015).

Finally, to investigate possibilities for the use of *L. casei* BL23 in a therapeutic approach, we assessed the strain *in vivo* for both local (*s.c.* administration at the same site of tumor injection) and therapeutic (i.e., after tumor challenge) effects. In this context, the presence of the bacteria significantly reduced tumor growth. However, the anti-cancer mechanisms of *s.c.* *L. casei* BL23 differed from those of *i.n.* administered bacteria. For example, *s.c*. injection of BL23 reduced NKp46 expression in tumor sections, but *i.n.* administration did not. In addition, preventive *i.n.* administration reduced tumor onset through a mechanism linked partially with local and systemic NK cell recruitment, while *s.c.* therapeutic administration had no effect on tumor onset, instead reducing tumor size via a recruitment of IL-2 and CD3 but NK-cell-independent mechanism.

## Conclusion

In conclusion, this study provides the first clues about the host molecular mechanisms involved in the anti-cancer effects of *L. casei* BL23. Our results may contribute to future efforts to develop probiotic-based food supplements for therapeutic applications in cancer treatment.

## Author Contributions

EJ and LB-H conceived and designed the study and wrote the manuscript. EJ conducted all the experiments. EJ, M-LM, and LB-H performed the data analysis. ET-M and FC provided technical help for the *in vivo* experiments. EJ, M-LM, PL, and LB-H discussed the experiments and results.

## Conflict of Interest Statement

The authors declare that the research was conducted in the absence of any commercial or financial relationships that could be construed as a potential conflict of interest.
