# Supplementary figures and images for: Elucidating the Immune-Related Mechanisms by Which Probiotic Strain Lactobacillus casei BL23 Displays Anti-tumoral Properties
Source: Front Microbiol. 2019 Jan 11;9:3281. doi: 10.3389/fmicb.2018.03281 (PMC6336716; doi:10.3389/fmicb.2018.03281)

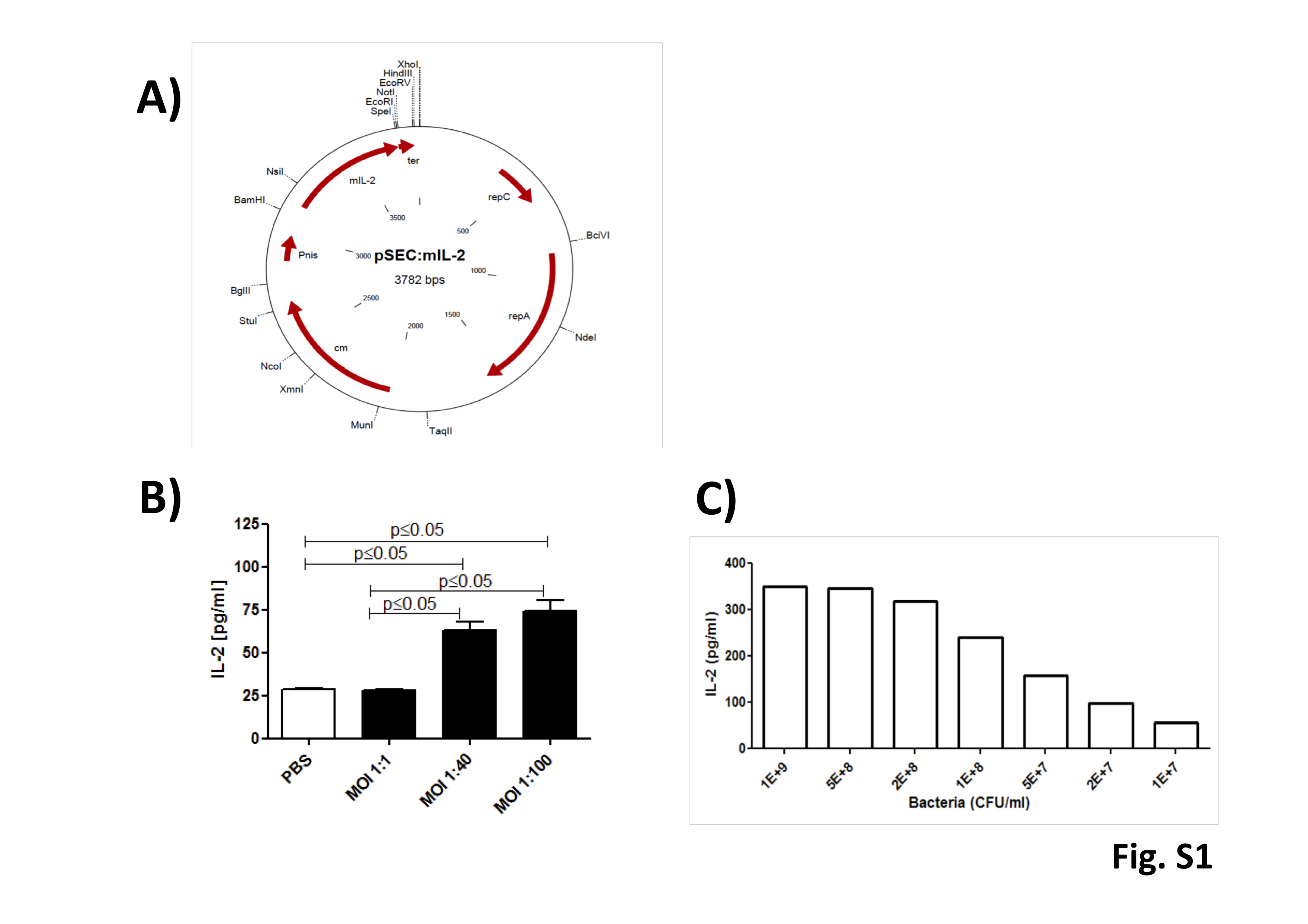

Supplement: Supplementary file 1 [file Image_1.TIFF]

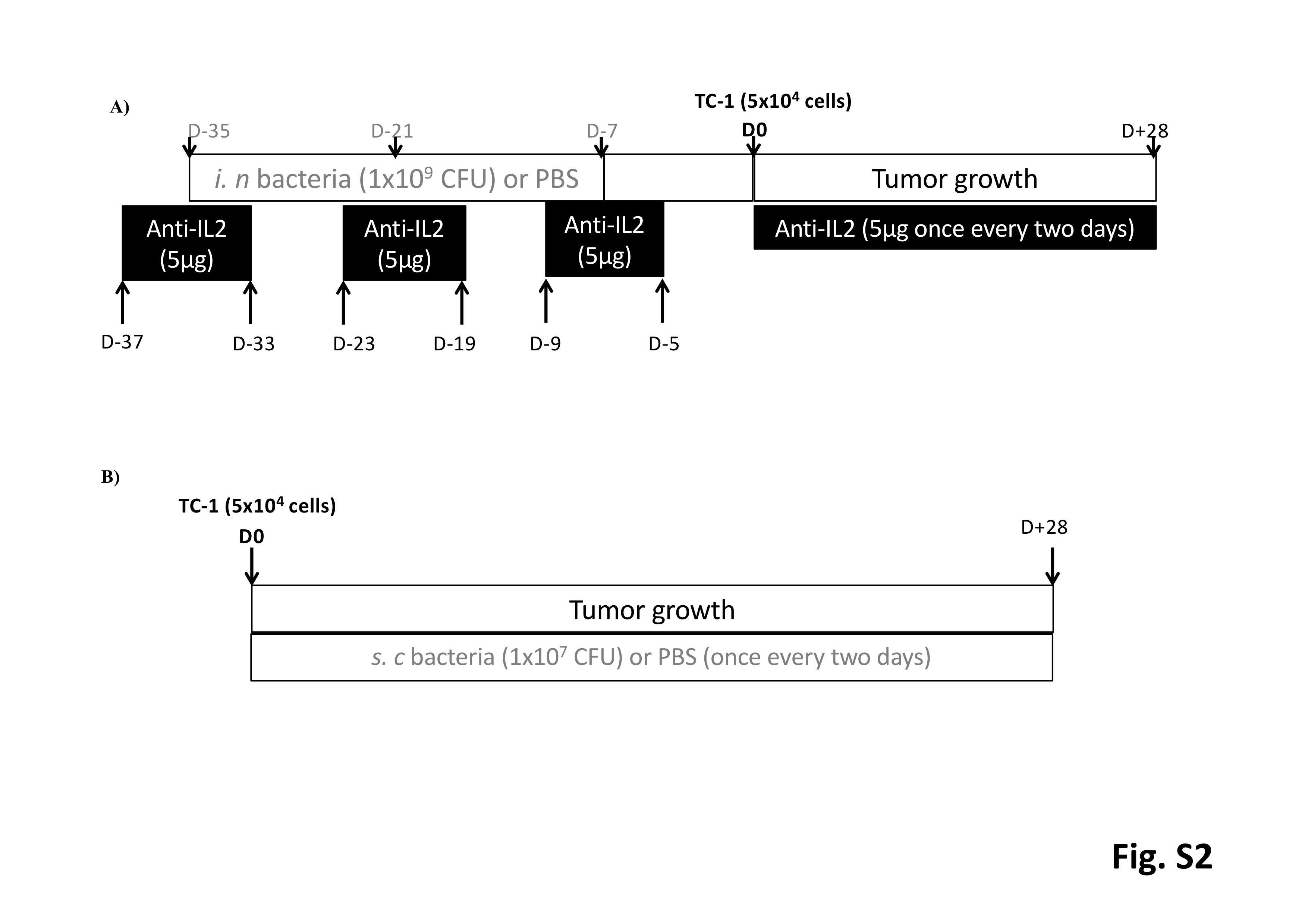

Supplement: Supplementary file 2 [file Image_2.TIFF]

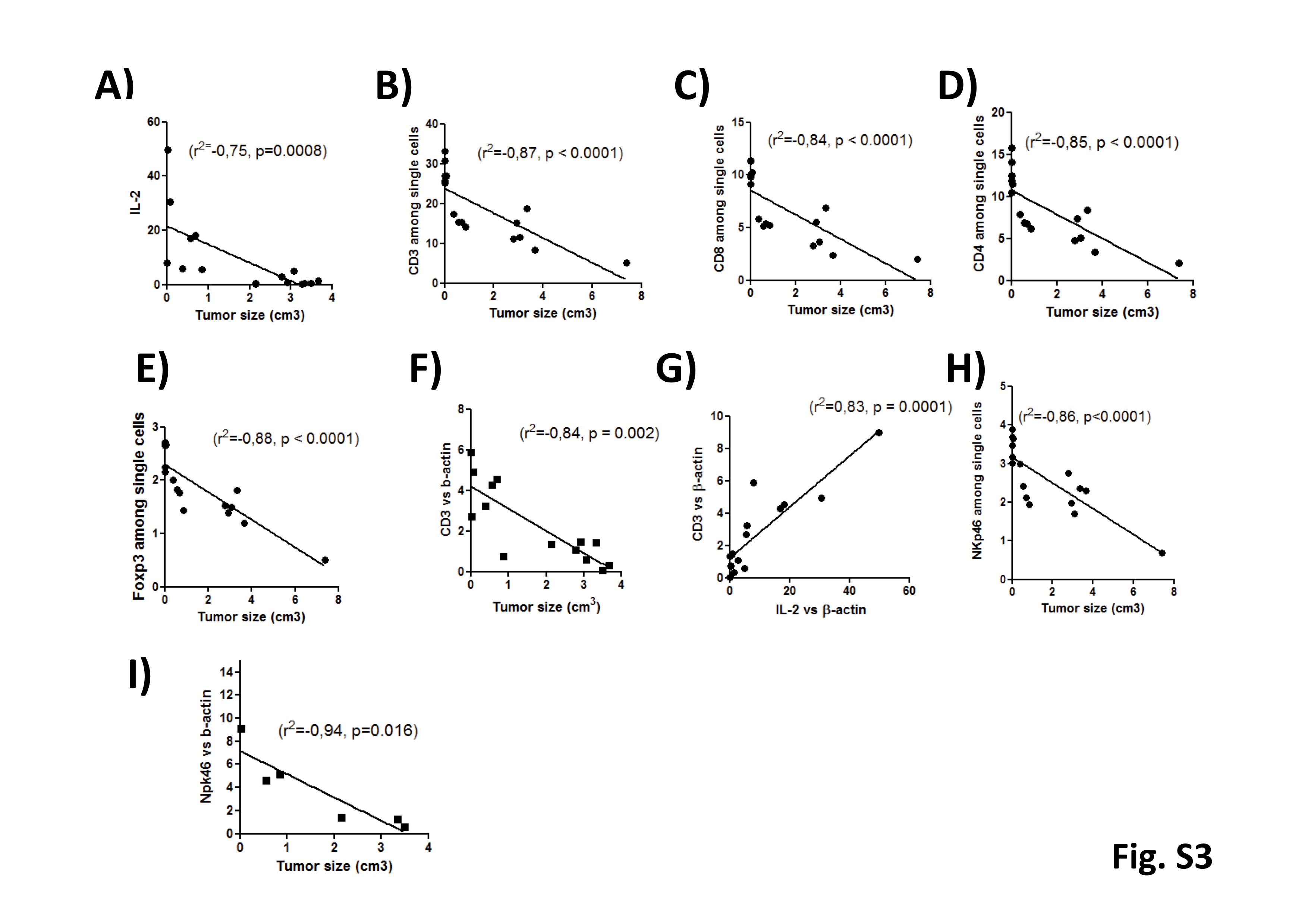

Supplement: Supplementary file 3 [file Image_3.TIFF]
